# A Rapid and Sensitive Microfluidics-Based Tool for Seroprevalence Immunity Assessment of COVID-19 and Vaccination-Induced Humoral Antibody Response at the Point of Care

**DOI:** 10.3390/bios12080621

**Published:** 2022-08-10

**Authors:** Kritika Srinivasan Rajsri, Michael P. McRae, Glennon W. Simmons, Nicolaos J. Christodoulides, Hanover Matz, Helen Dooley, Akiko Koide, Shohei Koide, John T. McDevitt

**Affiliations:** 1 Department of Molecular Pathobiology, Division of Biomaterials, Bioengineering Institute, New York University College of Dentistry, New York, NY 10010, USA; 2 Vilcek Institute of Graduate Biomedical Sciences, New York University School of Medicine, New York, NY 10016, USA; 3 Department of Microbiology and Immunology, Institute of Marine and Environmental Technology, University of Maryland School of Medicine, Baltimore, MD 21202, USA; 4 Department of Medicine, NYU Grossman School of Medicine, New York, NY 10016, USA; 5 Department of Biochemistry and Molecular Pharmacology, NYU Grossman School of Medicine, New York, NY 10016, USA; 6 Department of Chemical and Biomolecular Engineering, NYU Tandon School of Engineering, Brooklyn, NY 11201, USA

**Keywords:** COVID-19, SARS-CoV-2, seroprevalence, humoral immunity, diagnostics, point of care, microfluidics, clinical decision support tool

## Abstract

As of 8 August 2022, SARS-CoV-2, the causative agent of COVID-19, has infected over 585 million people and resulted in more than 6.42 million deaths worldwide. While approved SARS-CoV-2 spike (S) protein-based vaccines induce robust seroconversion in most individuals, dramatically reducing disease severity and the risk of hospitalization, poorer responses are observed in aged, immunocompromised individuals and patients with certain pre-existing health conditions. Further, it is difficult to predict the protection conferred through vaccination or previous infection against new viral variants of concern (VoC) as they emerge. In this context, a rapid quantitative point-of-care (POC) serological assay able to quantify circulating anti-SARS-CoV-2 antibodies would allow clinicians to make informed decisions on the timing of booster shots, permit researchers to measure the level of cross-reactive antibody against new VoC in a previously immunized and/or infected individual, and help assess appropriate convalescent plasma donors, among other applications. Utilizing a lab-on-a-chip ecosystem, we present proof of concept, optimization, and validation of a POC strategy to quantitate COVID-19 humoral protection. This platform covers the entire diagnostic timeline of the disease, seroconversion, and vaccination response spanning multiple doses of immunization in a single POC test. Our results demonstrate that this platform is rapid (~15 min) and quantitative for SARS-CoV-2-specific IgG detection.

## 1. Introduction

Vaccines and testing play parallel roles in the COVID-19 pandemic response and containment. Multiple studies have indicated that serum binding antibodies to SARS-CoV-2 spike receptor binding domain (RBD) and S proteins and neutralizing antibodies from COVID-19 convalescent plasma are maintained from 6 months to 1 year [1,2,3,4]. Additionally, studies have shown the presence of these antibodies correlates with protection against COVID-19 [5,6]. Conversely, other studies have shown that vaccination of otherwise healthy individuals elicited B cell antibody seroconversion, neutralizing antibodies, and T cell response-based immunity against reinfection and, importantly, protection against exhibiting severe COVID-19 for at least 6 months [7]. Although there is insufficient data on antibody titer thresholds that might indicate protection from infection, substantial evidence suggests that the immune response after infection plus vaccination typically leads to higher antibody titers [5,8,9,10]. These effects are not as robust in aging and immunocompromised individuals. Studies suggest that a smaller percentage of individuals seroconvert and sustain the neutralizing antibodies, making this population vulnerable to infection and potentially severe disease [11].

With at least 585 million people known to have been infected with SARS-CoV-2 worldwide to date and likely many more undetected/unreported cases, antibody responses could be measured across this population [12,13,14] to determine seroconversion against existing and rapidly mutating viral antigens. With the emergence of constantly mutating strains of COVID-19—some termed VoC—understanding infection history, the vaccination response, and the antibody response elicited becomes important and adds value to vaccine reception and dosage [15,16,17]. Thus, understanding and quantitatively screening the immune response is highly valuable, not just in immunocompromised individuals. Serosurveillance has insightful applications in diagnostics relating to COVID vaccination and/or infection elicited immune response [5,18].

There is currently significant interest in screening and understanding both humoral and cellular immunity [5,19], as our study also represents. The humoral or B cell antibody response demonstrates a significant aspect of the immunity conferred against SARS-CoV-2, being evaluated with significant importance [19,20,21,22,23]. Multiple labs are also assessing the cellular or T cell response to evaluate immunity induced by vaccination and/or infection [24,25]. These studies signify immense value to the understanding of immunity but have a high turnaround time, high cost, and need for skilled personnel to run an assay in a centralized lab involving stimulation of T cells and RNA purification steps prior to running an elaborate qPCR assessment of the T cell gene expression [24]. Importantly, all these immunity assessment studies represent time-consuming procedures not suitable for POC settings. Across studies, the S protein is shown to be highly immunogenic in eliciting a robust humoral response and is quite conserved among human coronaviruses [26,27]. Further, their interaction with the host cells has made them, and the less conserved RBD subunit, indispensable targets for diagnostics, vaccines, and therapeutic neutralizing antibodies [26]. Due to varying antibody levels based on individual immunity dynamics and the timeline of sample collection, the sensitivity of antibody tests may be variable, being more sensitive as the infection progresses and in individuals with established postinfection and postvaccine seroconversion [11,15,16]. Although IgM may have an early serological appearance, it usually has a low affinity to the target antigen. In contrast, IgG antibodies, which appear later, have higher titers and better affinities, making them better candidates for a detection test [28]. The enzyme-linked immunosorbent assay (ELISA) techniques commonly used in centralized clinical lab settings provide accurate and sensitive quantitative results but require long processing to result time, which delays onsite testing [29].

Over the last year, multiple microfluidics POC technologies have been reported, each quantifying viral proteins and antibodies with good precision and ease of use compared with traditional benchtop technologies [30]. Some integrated microfluidic detection systems have explored enhanced detection of SARS-CoV-2 nucleic acid compared with lateral flow assays, most based on the RT-LAMP technology [31,32,33]. Other POC devices with alternative microfluidics approaches—through fluorescence-based detection [34], impedance-based sensors [35], electrochemical-based sensors [36], and colorimetric detection [37]—have been reported in the past with application in infectious diseases and potential utility in the COVID-19 detection trail. Recently, some multiplexed SARS-CoV-2 antibody detection systems of interest have also been reported [38,39], although none of these have simultaneous rapid POC and quantitative capabilities.

In accordance with multiple studies demonstrating the significant value in assessing the SARS-CoV-2 antibody response in mitigating COVID-19 [19,22,40] and the ability to develop a rapidly (<20 min) quantitative and inexpensive antibody screen utilizing the anti-S RBD IgG at a POC setting, we have strived to develop an integrated lab-on-a-chip platform-based methodology that is rapid, quantitative and accurate while being easily accessible. This information, when available in quantitative, rapidly measurable POC settings, can help clinicians and scientists strategize evaluating the immune response after each vaccine dose and can also add insight toward the timing of subsequent boosts, ensure robust seroconversion in highly vulnerable patient populations and the selection of convalescent plasma donors and therapeutics to mitigate disease risk, and add essential value to vaccine perception and reception [41]. Critically, POC antibody tests are viable options for scaling up in community screening—being fast, easily accessible, and convenient.

We have recently published a general framework for implementing a POC clinical decision support system [42] that was adapted to the task of predicting mortality in cardiac patients with COVID-19 [43]. Additionally, a two-tiered system for evaluating COVID-19 prognosis in inpatient and outpatient settings was developed using data from a diverse population of patients across the New York City metropolitan area and externally validated using data from hospitals in Wuhan, China. Building upon this work, we present here the development and initial validation of a POC strategy for quantitative COVID-19 seroprevalence screening involving a SARS-CoV-2 antibody (IgG) test that covers the entire seroconversion timeline of the infection and vaccination response spanning across single and multidose vaccines—all over a single POC test. The work presented here is an important step forward toward completing the development of an integrated, accessible, and quantitative immunity screener, aiding the monitoring of seroconversion for epidemiological, preventive, and therapeutic applications at POC.

## 2. Materials and Methods

### 2.1. Immunoreagents and Buffers

All reagents used in the immunoassay were prepared with phosphate-buffered saline (PBS) buffer (Thermo Fisher Scientific Inc., Waltham, MA, USA). Additionally, a 3% bovine serum albumin (BSA) (Sigma-Aldrich, Burlington, MA, USA) solution was used for reagent stability and blocking nonspecific binding, and a sample carrier spiked in a dose-dependent manner with the analyte was also used. The RBD protein was produced in Expi293F cells transfected with the vector pCAGGS SARS-CoV-2 RBD (BEI Resources #NR-52309) following the methods of Stadlbauer et al., 2020, but using PEI as the transfection reagent, then supplementing the media with valproic acid as per Fang et al., 2017; protein purification was performed by gravity flow using Ni Sepharose excel resin (Cytiva, Marlborough, MA, USA). Antibody anti-Spike protein (RBD) (Sb#15), human IgG1-Fc fusion—Absolute antibody #ab02013-10.159, US and Canada, and nucleocapsid (NP) antigen (2019-nCoV—NP-His recombinant protein #40588-V08B, Beijing, China), and a goat antihuman IgG (H + L) cross-adsorbed secondary antibody Alexa fluor 488 (#A-11013, Invitrogen, Waltham, MA, USA) were procured. Human serum (Sigma-Aldrich; human male AB plasma, sterile-filtered) was utilized as a sample and tested spiked with or without the anti-RBD IgG antibody in different predetermined concentrations. Once acquired, the serum was tested to have not contained any pre-existing human COVID antibodies using clinical laboratory-based ELISA. For concerns of safety and toxicity of the human serum, each donor was tested and found nonreactive for HIV and hepatitis B and C antibodies by ELISA. Furthermore, the sterile-filtered human serum was heat inactivated at 56 °C for 30 min. Nevertheless, each reagent of human origin or otherwise used for this work was considered potentially infectious and handled safely with complete BSL-2 compliance.

### 2.2. Fabrication of the Microfluidic Cartridge

Assays were performed using prototype microfluidic cartridges and custom instrumentation and software, as described previously [44,45]. The base form of the cartridges, injection-molded polymethyl methacrylate (PMMA) prototype cartridges, were designed in-house and manufactured by Protolabs (USA). The top layers of the cartridges are constructed with 3M 9500PC double-sided adhesive and 3M AF4300 polyethylene terephthalate (3M Company, Maplewood, MN, USA) laminates, while the bottom layer utilizes Adhesives Research ARflow^®^ 93049 material, bound to the PMMA using a table-top hydraulic lab press (Carver, Inc). This specialty adhesive is hydrophilic and wicks the sample fluid into the cartridge via capillary action. Glass fiber pads (EMD Millipore, Burlington, MA, USA) were cut into 2 × 15 mm rectangles with SummaCut D75 and used to store detecting antibodies in the card. Agarose beads sensors were contained in a modular chip that features a 4 × 5 array of hexagon-shaped wells, cast using a UV-curable photopolymer (Norland Products Inc., East Windsor, NJ, USA) on a custom machined aluminum mold and cured for 30 min. Air vents were built into the card using hydrophobic SurePVDF membranes (EMD Millipore, USA) to mitigate bubble formation. To address the concerns of toxicity through the process of fabrication of these synthetic supplies, the entire process was performed in the presence of an industry-standard filter and fume extractor (BOFA^TM^). Additionally, all assembly was performed with the use of appropriate personal protective equipment (PPE).

### 2.3. Immunoassay, Imaging, and Analysis

Initial reagent validation and proof-of-concept assays were performed with Transwell membrane plates and inserts (Corning HTS Transwell—24-well permeable units w/0.4 and 3 μm polycarbonate membrane, USA) (Supplementary Figure S1). A volume of 10 µL of in-house activated conjugated agarose beads (250–300 µm size) was placed onto a polycarbonate membrane support. The phosphate-buffered saline (PBS) solution and 3% normal goat serum buffer sample (with or without anti-RBD antibody) were dispensed to the beads and incubated for 1 h while being mixed at low speed on a plate mixer. A wash was performed with 200 µL of PBS, and the beads were resuspended in the detection antibody and incubated before a final wash in PBS. After the beads settled on the membrane, they were imaged by fluorescence microscopy. The total assay time was about 4 h performed on a plate mixer. Imaging was performed using an Olympus BXFM fluorescent microscope. Images were analyzed using ImageJ (NIH) software using a region of interest (ROI) that encompassed the outer 10% of the bead sensor, calculating the corrected total cell/bead fluorescence method described in a protocol [46].

More integrated testing was completed on assay cartridges. Here, images were captured after each assay step using 5× magnification on a modified Olympus BXFM epifluorescent microscope (exposure at 1500 ms). Images were analyzed using software previously described [45], which averages the fluorescence intensity within an annular region of interest between the bead outer diameter and 90% of the bead radius. Calibration curves for the assays were completed and fit to a four-parameter logistic regression with MyCurveFit and SigmaPlot software. Unknown concentrations for samples were interpreted from the standard curve using spiked sampling methodology: 0 (Blank), 2.5, 12.8, 64, 320, and 1600, 8000, 40,000, and 200,000 ng of anti-RBD IgG/mL. Limit-of-detection (LOD) values were calculated using blank control replicates (average signal intensity plus three standard deviations). In the final stages of these studies, the complete set of chip-based measurements was completed using the fully integrated instrumentation

## 3. Results and Discussion

### 3.1. Assay Development

In order to produce lab-quality results in near-patient settings, it is necessary to have access to a testing platform that can complete all the sample processing, analyte capture, signal generation, and data processing within an integrated diagnostic platform. Using our previously described programmable bio-nano-chip (p-BNC) platform [47,48], we adapted this flexible tool to service the needs of a quantitative immunity screening device. Likewise, a quantitative microfluidics-based lab-on-a-chip POC antibody combination test was developed for the detection of anti-SARS-CoV-2 RBD IgG. Here, in-house fabricated agarose beads sensors (250–300 μm, 2% porosity), with the potential to host a variety of proteins and molecules, were utilized as the backbone for assay chemistry. Before moving these bead sensors into the final diagnostic cartridge, a surrogate method was used to optimize the bead sensor ensemble.

Likewise, the recombinant SARS-CoV-2 WA-1 RBD protein was conjugated in-house to the agarose bead sensors. Agarose beads hosting the antihuman IgG and nucleocapsid antigen were used as positive and negative controls, respectively. Initial reagent validation and proof of concept were performed with membrane plates and inserts (Supplementary Figure S1a) to serve as a surrogate test platform prior to the development of the microfluidic assays. The inserts allowed for the placement of the agarose beads onto the polycarbonate membrane while consistently being immersed in the sample buffer to allow the completion of different assay steps. Postwash beads were imaged under the fluorescence microscope by separating the inserts onto the imaging tray. The assay was performed in a sample (with and without antibody) titrated manner with appropriate controls (Supplementary Figure S1b). Images were captured on a fluorescent microscope on FITC, Cy5, and DAPI channels and stacked to generate image outputs used for analysis, followed by whole bead fluorescence measurements (Supplementary Figure S1c). A subsequent concentration vs. intensity curve was generated (Supplementary Figure S1d) to determine an initial detection range.

### 3.2. Assay Optimization and Instrumentation

**Figure 1:**
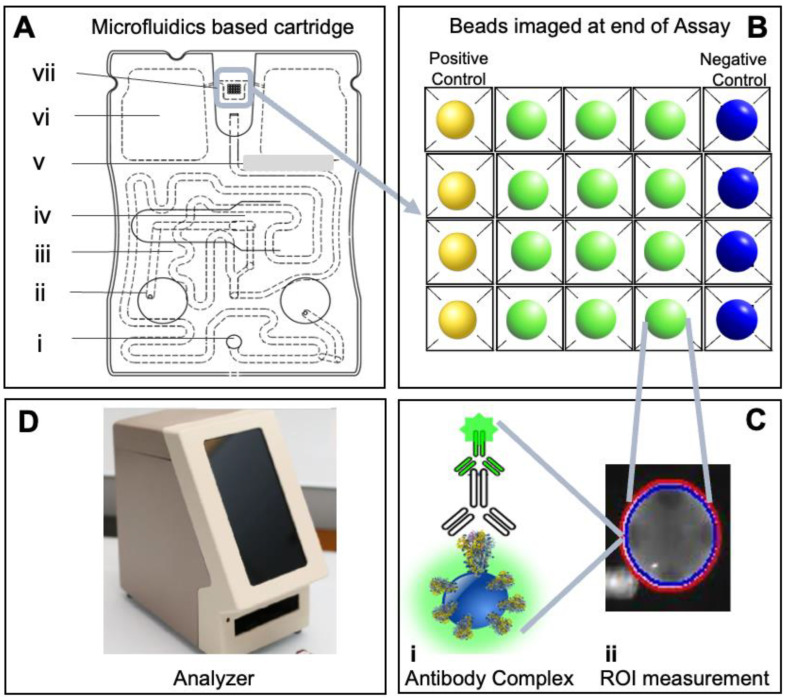
The point-of-care microfluidics-based combination antigen/antibody assay cartridge and analyzer. Illustration of assay cartridge (**A**) with sample input port (i), syringe pump adapter port (ii), sample loop (iii), glass fiber pad slot for detection reagent (iv), bubble trap (v), waste reservoir chamber (vi), and 4 × 5 bead array chamber (vii) shows an array of 20 programmable agarose bead sensors (**B**), with control and antibody capture beads imaged at steps 6 of the assay. The bead sensor serves as a high surface area substrate for developing programmable immunoassays for COVID-19 antibody detection and houses the immunocomplex (**C.i**). Image analysis for each individual bead sensor is performed by measuring 95% region of interest (ROI) on the bead (**C.ii**), capturing the many optimally stained and washed immune complexes formed over the bead. The integrated assay is completed in an automated fashion housed in the analyzer with optics, camera, actuator, blister actuators, integrated computer and display, and software to support automated analysis (**D**). While initial development work was completed with the aid of a commercial microscope, the final implementation was completed using the integrated instrumentation shown here.

Reagent and assay validation on the Transwell plates with inserts permitted the transition to validation and optimization of assays on the cartridge. The assay system is a fully integrated microfluidic network that functions as a portable diagnostic cartridge/reader system applicable for POC testing of a wide variety of clinical interest areas. The disposable injection-molded cartridge system consists of interconnected individual segments for parsing the various immunoassay steps, including sample and reagent introduction and delivery, fluid mixing, bubbles and debris removal, and dedicated analyte image capture through optical fluorescence signal readout. The cartridge functions through fluid/buffer delivery via three blisters and robust dual waste collection. The multilayer microfluidics immunoassay cartridge is a lab-on-a-chip platform featuring a network of microfluidic channels for sample delivery and metering, with detecting reagents embedded within the fluidic network and delivered through a series of flow steps. Additionally, mixers are patterned into the cartridge layer’s channels to ensure homogenous reagent delivery of fluids across the bead array (Figure 1A). Any bubbles entrained in flow are captured by the hydrophobic filter membrane material designed specifically as bubble trap structures. The analyte capture segment houses the 4 × 5 format bead array, with each vertically tapering well able to house a 250–300 μm agarose bead (Figure 1B), which accommodates the immunocomplex (Figure 1C). This design allows for connected fluid/buffer transport over the beads, allowing robust analyte capture across the 3D matrix of the porous agarose sensors and subsequent ease of quantitation. In addition, the reader features advanced optics, a complementary metal oxide semiconductor (CMOS) camera, a focus actuator, blister actuators, an external and internal code scanner, an integrated computer, display, and software to support automated analysis (Figure 1D). This evolution of this instrumentation system is summarized in Supplementary Figure S2.

This unified mini-sensor system allows for multiplexing biomarker detection and quantification of analyte and parallel immunoassay platforms while simultaneously helping translate tedious benchtop chemistry steps to POC. Each of these microporous ensemble beads hosts a network of ‘nano net’ structures that significantly increase the signal capture footprint and directly influence the assay timeline while being sensitive microsponge-like mediums. Recently, some groups have also shown significant applications of other nanotechnology and similar hydrogel-like materials; in diagnostics, vaccine delivery, and therapeutics, including polyanhydride, chitosan, poly(lactic-co-glycolic acid), and gold nanoparticles, among others [49]. Our novel biomimetic ‘nano net’ adapters resemble hydrogels and demonstrate a uniquely advanced application of nanotechnology in diagnostic applications, the other exception being gold nanoparticles capped with antisense oligonucleotide for detecting viral RNA in a sample [49].

### 3.3. Assay Execution and Integration

**Figure 2:**
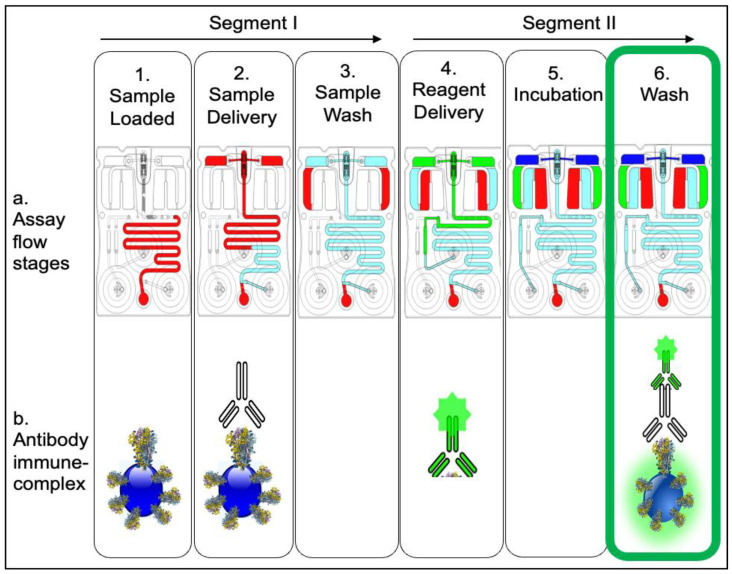
The COVID-19 antibody assay sequence. Step 1 shows the sample (+/− antibody) loaded to the cartridge input port, followed by sample delivery over the bead array through buffer flow via right blister (Step 2) and finished with a wash step (Step 3). Step 4 shows introduction of the antibody detection reagent conjugated to Alexa Fluor 488 that is eluted from the reagent pad over the bead array, followed by final incubation (Step 5) and final wash (Step 6) steps. In the presence of SARS-CoV-2 IgG1 antibody in the sample, the postassay completion image shows the antibody capture beads fluorescing as a result of the antibody immune complex formation (Step 6b).

The cartridge design allows for key immunoassay complexes to form on programmable 3D agarose bead sensors contained in the bead chip, integrated into the microfluidic environment, which aids optimal analyte capture and immunoassay completion and achieves low limits of detection. The spatial arrangement and redundancy of predefined beads and controls permit multiplexing and robust quantitative measurements. After the initial sample delivery to the bead array is completed via activation of flow pumps attached to the cartridge, subsequent incubation allows the capture of the analyte to the 3D bead sensor containing the capture reagent. The sample is delivered by pushing flow in through the right fluid input, which displaces the sample fluid to the bead sensors. Here the target-specific antibody present in the sample binds to the SARS-CoV-2 spike RBD bound to the bead sensor. After the sample is depleted, the composition of the flow grades to PBS buffer, automatically initiating a wash step to remove unbound analytes. In the next step, PBS buffer is delivered through the middle fluid input, which reconstitutes and releases the detecting reagent from the glass reagent pad. The detection reagent binds to the anti-SARS-CoV-2 antibody, which yields a fluorescent signal. Any unbound material is removed in a final wash step that removes all debris and analytes not specifically captured by the beads. All assay steps are summarized in Figure 2.

Here a 100 µL total sample volume (human serum spiked with or without the antibody) was introduced to the input port of the cartridge. The detection antibodies were deposited on the glass reagent pad. Agarose beads functionalized with capture reagents (recombinant SARS-CoV-2 WA-1 RBD protein) were placed in the 4 × 5 wells, with four-fold redundancy per analyte, permitting robust variance measurement and spatial identification while multiplexing. Positive (conjugated with antihuman IgG) and negative control beads (conjugated with NP antigen beads) were placed in the first and fifth columns, respectively. The positive and negative controls represent internal quality assurance and quality control beads in which the response parameters can be used as the basis for run rejection in the event of an error. A standard protocol was developed using automated fluid routing to control buffer flow across priming, sample/reagent delivery and incubation, and wash for sample (segment I: stages 1–3) and reagent (segment II: stages 4–6) introduction (Figure 2).

By actuating two different fluidic streams, approximately 350 μL of PBS was passed over the beads during each segment of the assay. Total time for each assay segment was approx. 5 min, and the total assay time was 15 min. For these initial experiments, the bead sensor priming, sample delivery, reagent incubation, and wash steps were performed using syringe pumps attached to the cartridge platform and imaged under the fluorescent microscope. Importantly, these lab-based research tools have been shown to produce nearly identical results as the fully integrated single push of the button point-of-care instrumentation described previously [44]. Integrated measurements that replicate the function and performance of the microscope and external syringe pump measurements were also completed for the studies presented here (see below). The formation of completed immune complexes (Figure 2—6b) over the 3D beads allows them to fluoresce in the FITC channel, captured under consistent imaging settings across samples.

**Figure 3:**
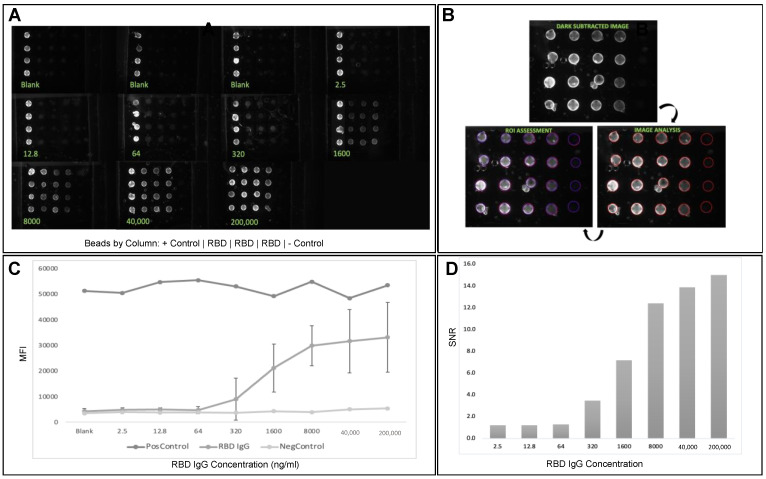
Fluorescent images show bead sensor arrangement and captured analyte, with variation in signal intensity at various concentrations (**A**). Image analysis was performed by measuring 95% ROI on sensor beads on a dark subtracted image captured during last stage of assay (**B**). The SARS-CoV-2 antibody concentration was assessed on the McDevitt lab point-of-care quantitative immunity scoring assay tool, and the dose curve here depicts a dynamic range between 10^0^ and 10^5^ (with potential to capture titers up to ~10^6^); all values depicted in the curve are represented by mean and error bars—standard deviation (**C**). Subsequently, signal-to-noise ratio (SNR) was calculated to indicate assay robustness and functionality (**D**).

After the initial validation and optimization studies were complete, standard curves for the antibody (anti-spike RBD) were completed with the five-fold serially diluted anti-RBD IgG-spiked sample buffer (Figure 3A), covering a range from very high Ig titers (200 μg IgG per ml) to very low titers (2 ng IgG per ml), as indicated in the recent literature [13,14,50]. Image analysis extracts the fluorescent intensity of the beads (Figure 3B), generating data sets to measure variances and signal-to-noise ratios. The resulting standard curves show increases in fluorescence intensity proportional to target analyte (IgG) concentration, with intra-assay precision ranging from 10–20%, encompassing a wide range required to cover the potential physiological range of anti-RBD antibody detection (Figure 3C). The limit-of-detection (LOD) calculations suggest 47 ng/mL for anti-SARS-CoV-2 IgG antibody detection, consistent with the lower biological threshold that would indicate positive SARS-CoV-2 antibody detection. Assay functionality was further demonstrated by the signal-to-noise ratio (SNR), showing a robust monotonic increase from low to high loads (SNR ~1 to 15, respectively) (Figure 3D).

**Figure 4:**
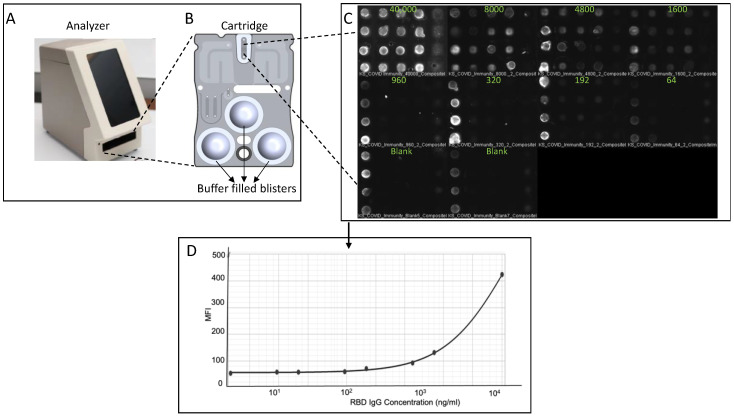
Initial proof of concept and reagent validation demonstration in an integrated POC format. The analyzer (**A**) with inserted blister mounted cartridge (**B**) allows for assay automation through integration of blister actuation, imaging, and data analysis, with visual engagement of each step through the touchscreen interface. Blank (no antibody) and positive (antibody present) sample assays were run on the integrated platform, and images were captured postassay run (**C**), followed by bead-to-bead fluorescent intensity measurement, data analysis followed by generation of dose–response curve (**D**).

Following the completion of the assay development and initial validation, it will be necessary to develop and clinically model the sample collection methodology that will be used within an integrated POC setting. A critical part of this process is to translate the assay parameters to completely integrated metrics, and we have most recently demonstrated a successful integration and validation of the immunity screening assay onto the existing p-BNC technology, as seen in Figure 4. The initial developments described here pave the pathway for the movement of immunity screening methods into real-world clinical practice. These integrated tests have the potential to reduce the time and costs associated with currently available approaches that target antibody detection via clinical lab-based tests, helping to address unmet needs in community settings and, in doing so, address concerns about the current gaps in testing associated with remote laboratory testing. This also reduces plastic waste and infectious material waste, which is better for the environment and reduces cost. In comparing this technology with other microfluidics-based POC tests [51], we demonstrate the advances and advantages of our technology and instrumentation in the ease of handling, including minimal training to run the assay, high sensitivity while being quantitative, and a potential capability to be coupled with smart devices and cloud services for ease of data transmission and tether patient history and other datasets for comprehensive diagnostic reporting, demonstrated in our previous work [43,52,53].

### 3.4. Development of Immunity Assessment

**Figure 5:**
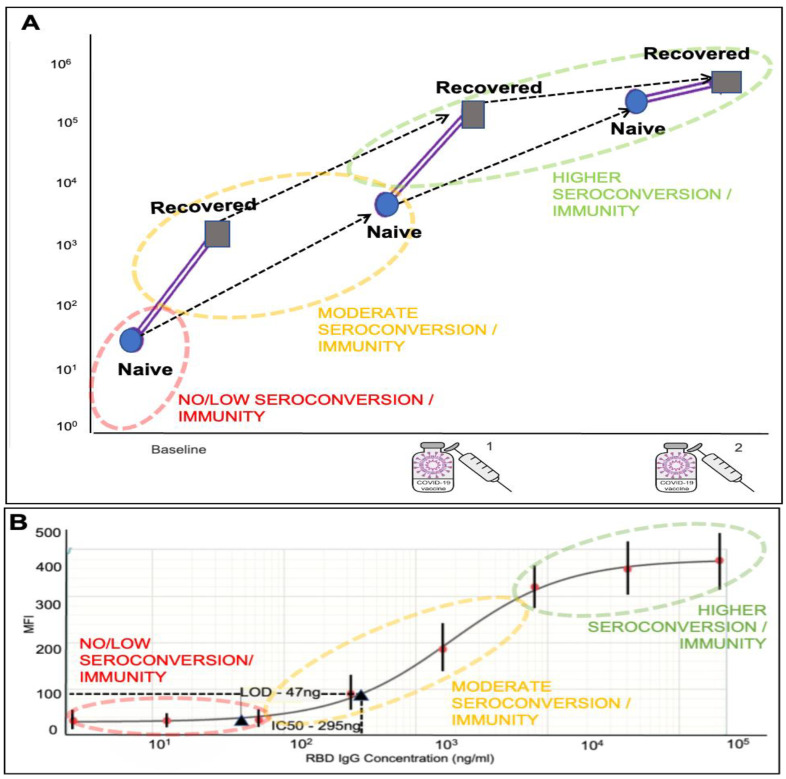
Median SARS-CoV-2 spike antibody titers compared in COVID-19 naïve (round blue end dots) vs. recovered square gray end dots) individuals—prevaccination and postvaccination, across six study data sets (Samanovic et al., Saadat et al., Stamatatos et al., Krammer et al., Rishi R. Goel et al., Joseph E. Ebinger et al.) (**A**). Panel (**B**) shows a standard curve from Figure 3C now depicting the SARS-CoV-2 antibody concentrations assessed on the McDevitt lab point-of-care quantitative immunity scoring assay tool, fitting the physiological range for anti-spike IgG antibody response, as seen above.

Having developed a robust assay and having shown its functionality in both lab-based and integrated instrumentation, our next step involved the exploration of how these assays might be used in the context of a clinical decision support tool. To explore this area, we also combined data sets from the recent literature that observe seroconversion responses, allowing us to model and compare antibody responses, including naïve and vaccinated data points (Figure 5A). This data helped generate a foundation for the immunity screening range, indicating individuals demonstrating no immunity at the lower end, partially immune moderate antibody response, and fully immune antibody titers at the highest range, corroborating with vaccination status and/or history of previous infection with vaccination adding a booster response. We have then overlaid these reference ranges to our standard curve, indicating the coverage of this range on our platform, as well as the ability to quantitatively report the antibody response (Figure 5B).

Backing the concept of a POC immunity screen, recent studies have made suggestions that individuals with prior infection exposure, leading to seroconversion, may achieve high antibody titers post one vaccine dosage compared with previously noninfected individuals [15]. In one study, the median baseline IgG antibody titers were 3–4 logs higher in the SARS-CoV-2 recovered compared with SARS-CoV-2 naïve individuals [54]. In the postvaccination, infection-recovered individuals were able to maintain a 1-to-2-log higher IgG titer than infection-naïve individuals only after the dose [13,55]. However, the booster (2nd) dose was able to raise the IgG tiers in infection-naïve individuals to the same levels as infection-recovered individuals. Surprisingly, the increase in IgG tiers was significantly improved in infection-naïve individuals after both doses but seemed to plateau after the first dose in infection-recovered subjects [12,13,14,54,55,56,57]. Other studies have shown similar results, with infection-recovered individuals showing higher antibody titers post the first dose but infection-naïve individuals requiring a second dose to improve their antibody titers to match that of the former (data points from these studies have been summarized and modeled in Figure 5A) [12,13,50,54,55,56].

Recent studies indicate that vaccination elicits distinct B cell and T cell responses between infection-recovered and -naïve subjects, pointing toward a robust antibody recall post the first dose of vaccine in the former group [54,55,58]. Thus, this indicates the importance for previously seropositive individuals to require vaccination to achieve significantly increased seroconversion with each vaccine dose. Serological testing alongside vaccination may add significant value in assessing prior and currently infected individuals who remain undiagnosed or undocumented [59], aiding in disease prevention, the assessment of potential convalescent plasma donors [60], and seroprevalence and serosurveillance studies [5]. Further, with a constant evolutionary race between the virus and host response, screening antibody titers in post vaccinated individuals may also provide insight into the efficacy and robust seroconversion against the newer VoC. Our tool can be tailored and multiplexed rapidly to sensitively screen antibody responses from the newer viral mutations. Antibody/seroconversion screening will potentially be utilized in upcoming months by epidemiological, healthcare, and various governing bodies, tailoring guidelines toward bringing societal normalcy and, importantly, managing immunocompromised and aging individuals [12,13,14,50,61].

The POC tools with enhanced diagnostic accuracy could be used in easily accessible settings with the potential to have a dramatic influence on community screening and immunity profiling alongside safe management of COVID-19 spread. With the advent of COVID-19 vaccinations playing a role in disease prevention, robust immune responses in individuals postvaccination will not only play a protective role individually but also achieve herd immunity [5]. Continuous improvements in diagnostic tests are essential for rapid detection of patients, surveillance, and healthcare preparedness, both in high- and low-resource settings. While these kits are not currently available for widespread use, public and private organizations worldwide are working on prototypes, with over 50 currently in development [62]. To date, these new diagnostic tests have been developed outside an integrated screening procedure. The development and customization of diagnostic tests is a key priority alongside its use with gated patient screening and risk-based triage procedures. None of the existing diagnostic tests cover the initial screening process, comprehensive POC diagnostic testing, as well as immunity screening for community risk assessment.

A significant challenge faced in the development of various tests in the diagnostic menu, including antibody-specific assays, relates to the highly evolving viral strain and cross-reactivity between other coronaviruses (SARS-CoV, MERS), as well as lowered sensitivity due to epitope changes/mismatches [63]. These issues can be mitigated through optimization of reagent sources, subtypes, blocking strategies, assay flow rates, and volumes. Further, limitations of this testing strategy include obtaining negative results in patients during the early incubation period of an ongoing infection later becoming infectious. Cost, complexity, and supply chain shortages are bottlenecks for scaling SARS-CoV-2 immunity screening. This work serves to demonstrate a new technology for assessing antibodies to screen immunity associated with COVID-19 and vaccination. This promising tool can be implemented in high-risk settings requiring rapid, cost-effective, convenient, and accurate screening results. The next steps of this study will explore the analytical performance of this system and will involve the assessment of qualitative performance (sensitivity and specificity) and blinded validation of the combinatorial format with the “spiked sera” and “patient samples” compared with control samples, confirmed by RT-PCR and lab-based serological testing methods. Data from these studies will be collected to the end of precision studies and clinical validation, including logistic regression modeling, to generate ROC curves and correlation assessment. Additionally, longitudinal, multi-time point assessments will be made to assess changes in immunity across horizontal timelines. Establishing sensitivity and specificity for POC tests is a critical step in establishing their efficiency as a diagnostic screening tool. Either in the current assay state, progressing toward initial clinical validation, or introducing evidence-based changes into the assay format (that is, swapping reagents or adding additional data points for improving testing efficiency) would entail rigorous reagent assessment through validation, optimization, and quantitation—requiring a robust supply chain of working reagents. The use of a programmable diagnostics platform, as described in this study already, allows for an agile approach well suited for this dynamically changing infectious disease target.

## 4. Conclusions

In closing, we have developed a novel application of a programmable microfluidic platform for rapid, accurate, and quantitative detection of SARS-CoV-2-specific humoral response to assess seroprevalence and humoral immunity. These new integrated medical microdevice systems have the potential to help profile the immune status of individuals, informing public health strategies: risk stratification of individuals, particularly in the aging and immune-compromised population, to isolate the variables contributing to increased morbidity and mortality, rational designing and deployment of vaccine doses, rapid identification of a convalescent plasma donor, a better understanding of the mechanisms of immunity, facilitate individual or institutional decision-making, and epidemiological monitoring of seroconversion. We believe that understanding the specific role of the humoral response to SARS-CoV-2, gathered in a rapid quantitative POC setting, can complement the lab-based cellular and humoral immune response assessments to further mitigate our vulnerability to the virus.

## Data Availability

Data is contained within the article and Supplementary Material. The data presented in this study are available across the Figures and Supplementary Figure S1 in this manuscript. Further information and data presented in this study are available on request from the corresponding author.

## Supplementary Materials

The following supporting information can be downloaded at: https://www.mdpi.com/article/10.3390/bios12080621/s1, Figure S1: Immunoassay on Transwell plates with inserts (a) were performed for initial proof of concept and reagent validation studies. Antibody titration was also performed alongside controls and imaged under a fluorescent microscope (b). The images were examined with the ImageJ (NIH) software and individual beads analyzed with whole bead fluorescence intensity measurement method (c), and intensity vs. concentration dataset generated (d). Figure S2: This illustration shows the evolution of instrumentation and the fluidics system that were used to capture the measurements completed in this manuscript.
