# Supplementary material for: A Rapid and Sensitive Microfluidics-Based Tool for Seroprevalence Immunity Assessment of COVID-19 and Vaccination-Induced Humoral Antibody Response at the Point of Care
Source: Biosensors (Basel). 2022 Aug 10;12(8):621. doi: 10.3390/bios12080621 (PMC9405565; doi:10.3390/bios12080621)
Supplement: Supplementary file 1 [file biosensors-12-00621-s001.zip › biosensors-1832974-supplementary.pdf]

SUPPLEMENTARY FIGURES

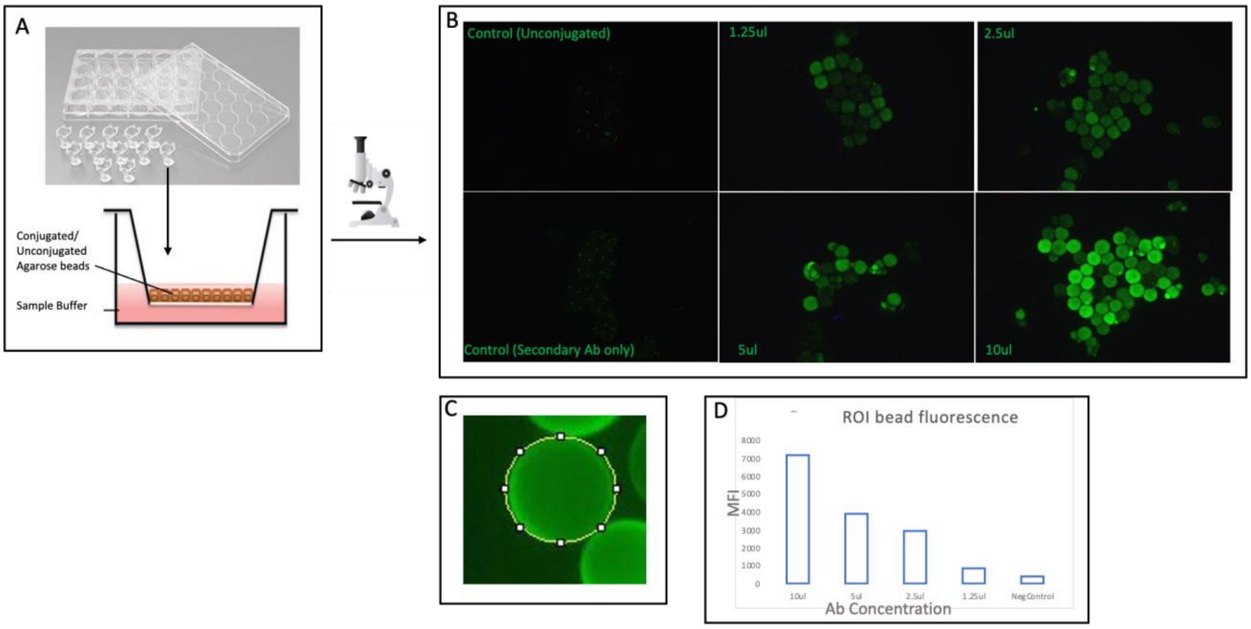

Figure S1. Immunoassay on Transwell plates with inserts (a) were performed for initial proof of concept and reagent validation studies. Antibody titration was also performed alongside controls and imaged under a fluorescent microscope (b). The images were examined with the ImageJ (NIH) software and individual beads analyzed with whole bead fluorescence intensity measurement method (c), and intensity vs. concentration dataset generated (d).

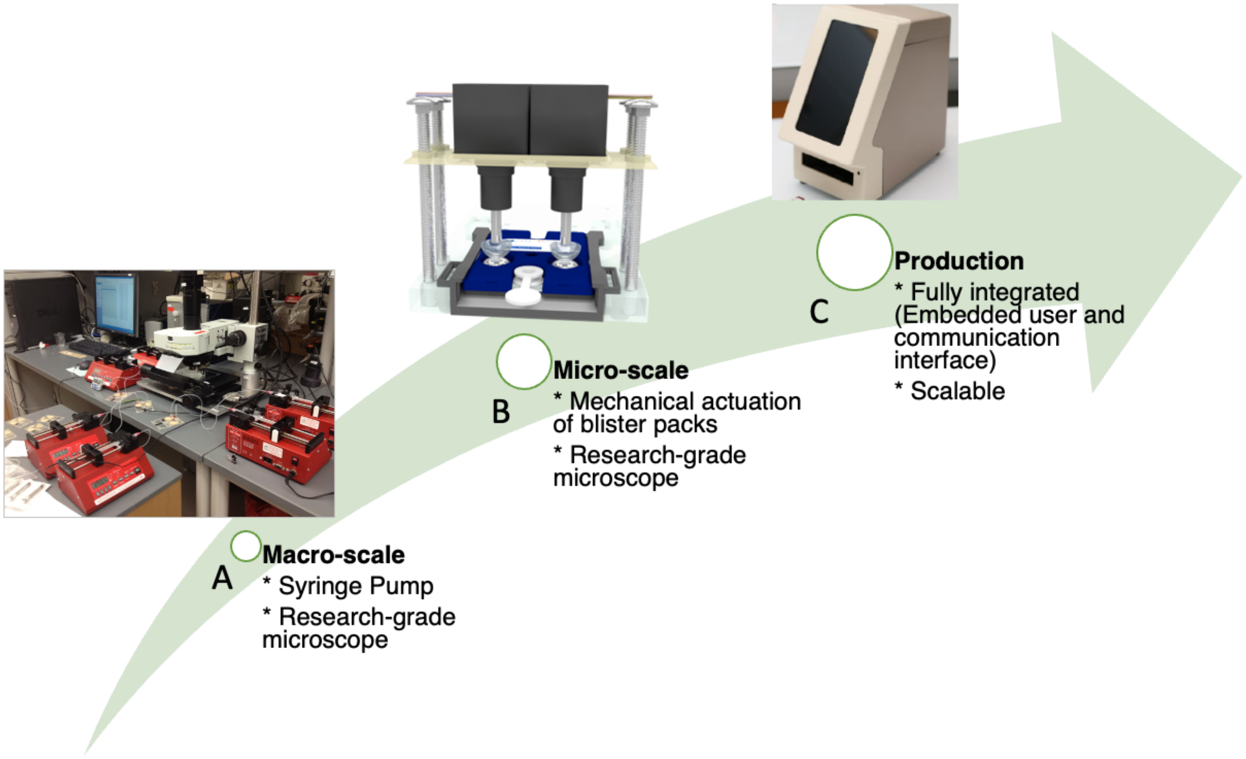

Figure S2. This illustration shows the evolution of instrumentation and the fluidics system that were used to capture the measurements completed in this manuscript.
